# Response Monitoring with [^18^F]FLT PET and Diffusion-Weighted MRI After Cytotoxic 5-FU Treatment in an Experimental Rat Model for Colorectal Liver Metastases

**DOI:** 10.1007/s11307-016-1021-2

**Published:** 2016-10-31

**Authors:** Sandra Heskamp, Linda Heijmen, Danny Gerrits, Janneke D. M. Molkenboer-Kuenen, Edwin G. W. ter Voert, Kathrin Heinzmann, Davina J. Honess, Donna-Michelle Smith, John R. Griffiths, Sabrina Doblas, Ralph Sinkus, Peter Laverman, Wim J. G. Oyen, Arend Heerschap, Otto C. Boerman

**Affiliations:** 1 Department of Radiology and Nuclear Medicine, Radboud University Medical Center, Nijmegen, The Netherlands; 2 Cancer Research UK Cambridge Institute, University of Cambridge, Cambridge, UK; 3 LBI, CRI – UMR 1149 Inserm, Université Paris Diderot, Paris, France; 4 BHF Centre of Excellence, Division of Imaging Sciences and Biomedical Engineering, King’s College London, King’s Health Partners, St. Thomas’ Hospital, London, SE1 7EH UK

**Keywords:** 5-Fluorouracil, [^18^F]FLT PET, Diffusion-weighted MRI, Colorectal cancer, Response monitoring

## Abstract

**Purpose:**

The aim of the study was to investigate the potential of diffusion-weighted magnetic resonance imaging (DW-MRI) and 3′-dexoy-3′-[^18^F]fluorothymidine ([^18^F]FLT) positron emission tomography (PET) as early biomarkers of treatment response of 5-fluorouracil (5-FU) in a syngeneic rat model of colorectal cancer liver metastases.

**Procedures:**

Wag/Rij rats with intrahepatic syngeneic CC531 tumors were treated with 5-FU (15, 30, or 60 mg/kg in weekly intervals). Before treatment and at days 1, 3, 7, and 14 after treatment rats underwent DW-MRI and [^18^F]FLT PET. Tumors were analyzed immunohistochemically for Ki67, TK1, and ENT1 expression.

**Results:**

5-FU inhibited the growth of CC531 tumors in a dose-dependent manner. Immunohistochemical analysis did not show significant changes in Ki67, TK1, and ENT1 expression. However, [^18^F]FLT SUV_mean_ and SUV_max_ were significantly increased at days 4 and 7 after treatment with 5-FU (60 mg/kg) and returned to baseline at day 14 (SUV_max_ at days −1, 4, 7, and 14 was 1.1 ± 0.1, 2.3 ± 0.5, 2.3 ± 0.6, and 1.5 ± 0.4, respectively). No changes in [^18^F]FLT uptake were observed in the nontreated animals. Furthermore, the apparent diffusion coefficient (ADC_mean_) did not change in 5-FU-treated rats compared to untreated rats.

**Conclusion:**

This study suggests that 5-FU treatment induces a flare in [^18^F]FLT uptake of responsive CC531 tumors in the liver, while the ADC_mean_ did not change significantly. Future studies in larger groups are warranted to further investigate whether [^18^F]FLT PET can discriminate between disease progression and treatment response.

**Electronic supplementary material:**

The online version of this article (doi:10.1007/s11307-016-1021-2) contains supplementary material, which is available to authorized users.

## Introduction

During the last decade, treatment of patients with colorectal cancer (CRC) liver metastases has significantly improved, mainly due to improved surgical techniques, more effective chemotherapeutic agents, and new targeted drugs [1, 2]. However, the backbone of treatment still consists of chemotherapy with fluoropyrimidines: intravenous 5-fluorouracil (5-FU) or oral fluoropyrimidines. These agents are used in various combinations and schedules, such as 5-FU in combination with leucovorin (LV), irinotecan (FOLFIRI), or oxaliplatin (FOLFOX), and have significantly improved survival. Nonetheless, ultimately most patients will develop progressive disease [3]. Currently, there are no adequate biomarkers or early response monitoring techniques that predict response to 5-FU-containing chemotherapy schedules.

Two imaging techniques that have potential as early imaging biomarkers for therapy response or resistance are 3′-deoxy-3′-[^18^F]fluorothymidine ([^18^F]FLT) positron emission tomography (PET) and diffusion-weighted magnetic resonance imaging (DW-MRI). [^18^F]FLT is a thymidine analog, and its uptake in tumor cells is primarily mediated by the equilibrative nucleoside transporter 1 (ENT1) [4]. Upon internalization, [^18^F]FLT is phosphorylated by thymidine kinase (TK1) and consequently trapped in the tumor cell. Elevated TK1 expression is observed during cell proliferation, and therefore, [^18^F]FLT PET is a potential marker for tumor cell proliferation [5].

Apart from measuring tumor cell proliferation, early response can be monitored by imaging biomarkers that detect tumor cell death. DW-MRI measures Brownian motion of water molecules, and the quantitative parameter for this motion in tissue is the apparent diffusion coefficient (ADC). Restricted diffusion, reflected by a low ADC, is found in highly cellular tissue with intact cell membranes, such as tumors. Less restricted diffusion, a high ADC, is found in tissue with a low cellularity and disrupted cell membranes, such as necrotic tissue. Therefore, DW-MRI can potentially be used to characterize cellular integrity and chemotherapy-induced cell death [6].

The aim of our study was to determine whether [^18^F]FLT PET and DW-MRI have potential for early response monitoring after cytotoxic 5-FU treatment. For that purpose, we induced colorectal tumors in the liver of Wag/Rij rats, as an experimental model for liver metastasis of CRC, and studied the effect of 5-FU on tumor [^18^F]FLT uptake and ADC.

## Material and Methods

### Cell Culture and Radiolabeling

The rat colon carcinoma cell line CC531 was derived from Wag/Rij rats exposed to 1,2-dimethyl-hydrazine and was cultured as described previously [7, 8]. [^18^F]FLT was obtained from the Department of Nuclear Medicine and PET Research, VU University Medical Centre, Amsterdam, The Netherlands, and was produced as described previously [9, 10]. Radiochemical purity of [^18^F]FLT exceeded 95 %. The monoclonal antibody MG1, purchased from Antibodies for Research Applications BV (Gouda, The Netherlands), recognizes a 80 kDa cell surface antigen on CC531 cells and was radiolabeled with In-111 as described previously [11, 12].

### Effect of 5-FU on [^18^F]FLT Uptake of CC531 Cells *In Vitro*

CC531 tumor cells were allowed to adhere overnight in six-well plates and were subsequently cultured in the presence of 10 μM 5-FU for different periods of time (6–72 h). Since 5-FU is generally administered in combination with other chemotherapeutic agents, we also treated cells with oxaliplatin (1 μM), or 5-FU (10 μM) + oxaliplatin (1 μM), or 5-FU (10 μM) + oxaliplatin (1 μM) + leucovorin (2 μM). Leucovorin enhances the effect of 5-FU by inhibiting thymidylate synthase (TS). To measure the effect of 5-FU on [^18^F]FLT uptake, 0.1 MBq of [^18^F]FLT was added to each well, followed by incubation for 2 h at 37 °C in a CO_2_ incubator. At the end of incubation, cells were washed once with phosphate-buffered saline, lysed in 0.1 M NaOH, and the [^18^F]FLT uptake was measured in a gamma counter. Separate wells were used to determine the protein concentration as a measure for the number of cells per well (BCA Protein Assay Reagent, Thermo Scientific).

### Animal Experiments

Experiments were carried out in Wag/Rij rats, 6 to 8 weeks old, obtained from Charles River Laboratories (Sulzfeld, Germany). Experiments were carried out in female rats, unless stated otherwise. The animals were acclimatized to laboratory conditions for 1 week prior to experimental use and housed under nonsterile, standard laboratory conditions (temperature, 20–24 °C; relative humidity, 50–60 %; 12 h light/12 h dark) on sawdust in individually ventilated cages (two or three rats per cage) with free access to animal chow (Snif Voer, Soest, The Netherlands) and water. All experiments were conducted in accordance with the principles laid out by the revised Dutch Act on Animal Experimentation (WOD) and approved by the institutional Animal Welfare Committee of the Radboud University Nijmegen. During all experiments, general health and body weight of the rats were monitored. CC531 tumors cells were injected subcapsularly in the liver as described previously [13].

### Dose Optimization 5-FU Therapy and SPECT/CT with [^111^In]MG1

Four groups of five male Wag/Rij rats with intrahepatic CC531 tumors were treated with four cycles of 5-FU at different dose levels (vehicle, 15, 30, or 60 mg/kg (in 0.2 ml saline)) to determine the optimal dose that inhibits growth of CC531 tumors, without causing severe side effects. To monitor the tumor growth of the intrahepatic tumors noninvasively, two rats per group underwent SPECT/CT imaging at days 5, 12, and 19 after start of treatment (same rats per group were scanned at each time point) using In-111-labeled MG1 antibody as a tracer to visualize tumors [14]. Three days prior to SPECT/CT, rats were injected intravenously with 20 MBq (17–19 μg) [^111^In]MG1. SPECT/CT scans were acquired with the U-SPECT-II/CT (MILabs, Utrecht, The Netherlands). Rats were scanned under general anesthesia (isoflurane/O_2_) for 50 min using the 1.0-mm diameter rat collimator tube, followed by a CT scan (spatial resolution 160 μm, 65 kV, 615 μA) for anatomical reference. Scans were reconstructed with MILabs reconstruction software, using an ordered-subset expectation maximization algorithm, with a voxel size of 0.75 mm. SPECT/CT scans were analyzed, and maximum intensity projections (MIPs) were created using the Inveon Research Workplace software (IRW, version 4.1). At day 23, all rats were euthanized by CO_2_/O_2_ asphyxation and the liver was examined macroscopically for tumor growth. CC531 tumors were excised and weighed. Plasma, liver, and tumor samples of two rats from each group were collected to determine the *in vivo* thymidine levels. For this, a liquid-chromatography—mass spectrometry (LC-MS/MS) method for the quantitative analysis of thymidine in tumor tissue homogenate was developed by the PK/Bioanalytics Core Facility at the CRUK Cambridge Institute [15, 16]. The biodistribution of In-111-labeled MG1 was determined after dissection as described previously [13].

### Treatment Response Monitoring Studies

Intrahepatic liver tumors were induced in ten groups of Wag/Rij rats (*n* = 7 per group). After 2 weeks, rats underwent baseline T2-weighted and DW MRI (day −1). The next day (day 0), rats received a single i.p. injection (5-FU at 60 or 30 mg/kg or vehicle). At day 1, 3, or 7 after treatment, a follow-up DW-MRI was acquired. After the DW MRI, rats were injected with [^18^F]FLT and were euthanized 60 min later by CO_2_/O_2_ asphyxation. Blood, tumor, and normal liver were collected to determine the [^18^F]FLT uptake *ex vivo*. Tumor tissue was fixed in 4 % formaldehyde and embedded in paraffin for immunohistochemical analysis. Rats were excluded from the analysis if no tumor was visible on the baseline T2-weighted image or when DWI or [^18^F]FLT PET acquisition failed because of technical reasons.

To study the effect of 5-FU on [^18^F]FLT PET in more detail, an additional response monitoring study was performed in two groups of seven rats with intrahepatic liver tumors. Rats underwent a baseline [^18^F]FLT PET (day −1), followed by 5-FU treatment (60 mg/kg or vehicle) at days 0 and 7. Follow-up [^18^F]FLT PET scans were acquired at days 1, 4, 7, and 14 after the first administration of 5-FU or vehicle. After the last scan, rats were euthanized and blood, tumor, and normal liver were collected to determine the [^18^F]FLT uptake. Tumor tissue was fixed and paraffin embedded for immunohistochemical analysis.

### DW-MRI

DW-MRI was performed at 11.7T on a Bruker Biospec MR system under Paravision 5.1 using a Bruker Mouse Body Volume Coil. The outer diameter of the coil is 75 mm, and the inner diameter is 40 mm, which is suited to fit small rats. Anesthesia was induced with 4 % isoflurane, and rats were maintained under anesthesia with 2 % isoflurane; the respiratory rate of the rats was monitored and kept constant at approximately 40 breaths/min, and temperature was maintained at 37 °C. For morphological assessment, a T2-weighted MR image was acquired with slices covering the whole tumor area. The slice with the largest tumor diameter was chosen for DW MRI. DW MRI acquisition was respiratory gated and followed a spin-echo EPI scheme with fat suppression, TE/TR of 16/2000 ms, 4 averages, 23 segments, b-values 0, 150, 300, and 600 s/mm^2^, slice thickness 2 mm, FoV 5 cm, matrix 128 × 128. ADC maps were calculated using b = 0, 150, 300, and 600 s/mm^2^ images. Tumor ROIs were defined manually on the b = 0 images and then copied on the ADC maps. ADC_mean_, ADC_median_, and 25th and 75th percentile values were calculated.

### [^18^F]FLT PET

[^18^F]FLT PET scans were acquired using the Inveon PET/CT system (Siemens Preclinical Solutions, Knoxville, TN) with an intrinsic spatial resolution of 1.5 mm [17]. Rats were injected intravenously with 10–12 MBq [^18^F]FLT and 50 min postinjection, anesthesia was induced with 4 % isoflurane in air. Rats were maintained under anesthesia with 2 % isoflurane. At 60 min postinjection, a PET emission scan was acquired for 15 min (60–75 min postinjection), followed by a Co-57 transmission scan of 3 min. Scans were reconstructed using the Inveon Acquisition Workplace software (version 1.5, Siemens Preclinical Solutions), using an ordered-set expectation maximization 3D maximum *a posteriori* algorithm with the following parameters: matrix, 256 × 256 × 159; pixel size, 0.43 × 0.43 × 0.8 mm^3^; and β-value of 1.5 with uniform variance. A 3D isocontour was set at 41 % of the maximum pixel value adapted for background (normal liver) to determine pretreatment and posttreatment SUV_mean_ and SUV_max_ [18]. To determine the SUV_max_ in bone marrow, a 3D volume of interest was drawn around the left femur and subsequently, an isocontour was set at 50 % of the maximum pixel value.

### Immunohistochemistry

Antibodies against Ki67 (RM-9106-S1, Thermo Scientific), TK1 (GTX113281, Genetext), and ENT1 (ab182023, Abcam) were used to determine expression of the respective antigens on paraffin-embedded tumor sections (4–7 tumor sections per group). Antigen retrieval was performed for 10 min at 99 °C in 10 mM sodium citrate pH 6.0 (Ki67 and TK1) or 10 mM TRIS, 1 mM EDTA, 0.05 % Tween, pH 9.0 (ENT1). Endogenous peroxidase activity was blocked with 3 % H_2_O_2_, and nonspecific binding was blocked by incubation with normal swine serum (Ki67 and TK1) or normal goat serum and avidin/biotin blocking (SP-2001, Vector, ENT1). After incubation with the primary antibody, Ki67 and TK1-stained tumor sections were incubated with a peroxidase-conjugated secondary antibody and ENT1-stained sections with a biotinylated secondary antibody, followed by incubation with an avidin-biotin-enzyme complex (Vector Laboratories, Burlingame, CA). Finally, 3,3′-diaminobenzidine (DAB) was used to develop the staining of the tumor sections.

### Statistical Analysis

Statistical analyses were performed using IBM SPSS Statistics version 20.0 (Chicago, IL). The nonparametric, independent samples, Kruskal-Wallis test was used to test differences in *in vitro* [^18^F]FLT uptake and tumor load after 5-FU treatment. Differences in ADC_mean_, SUV_mean_, and SUV_max_ were tested for significance using the related-samples Wilcoxon-signed rank test or nonrelated samples Mann-Whitney *U* test. All tests were two-sided, and a *p* <0.05 was considered significant.

## Results

### 5-FU Increases the Uptake of [^18^F]FLT of CC531 Cells *In Vitro*

**Fig. 1.**
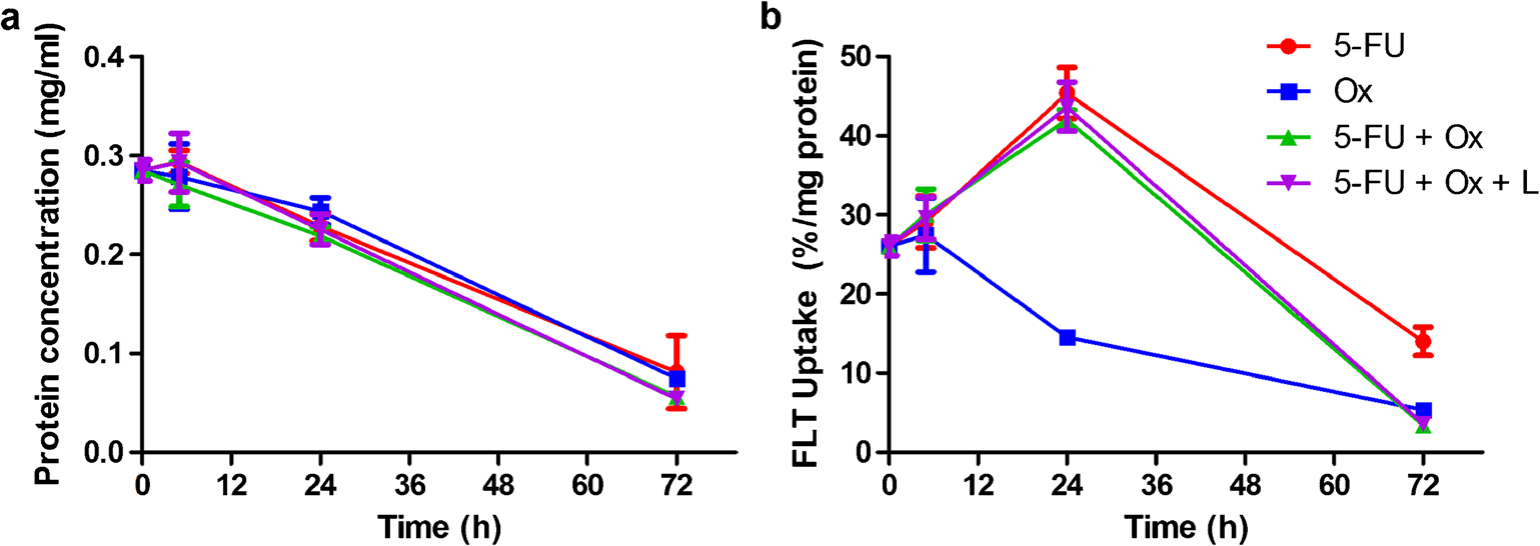
**a** Protein concentration of CC531 cell lysates and **b** [^18^F]FLT uptake of CC531 cells cultured with 5-FU (10 μM), oxaliplatin (Ox, 1 μM), or a combination of 5-FU with Ox with or without leucovorin (L, 2 μM). Uptake was measured as percentage of the added radioactivity per milligram cell protein.

*In vitro*, 5-FU (10 μM), oxaliplatin (1 μM), as well as the combination of agents, inhibited the growth of CC531 cells (Fig. 1a). Oxaliplatin alone significantly reduced the uptake of [^18^F]FLT at 24 h (−44 ± 3.4 %) and 72 h (−79 ± 0.5 %) after start of treatment (*p* = 0.024), while 5-FU alone significantly increased [^18^F]FLT uptake after 24 h with 75 ± 12.2 % (*p* = 0.022). The 5-FU induced increase in [^18^F]FLT uptake was not affected by adding oxaliplatin (uptake increased with 62 ± 5 %, Fig. 1b).

### 5-FU Inhibits Growth of CC531 Liver Tumors

**Fig. 2.**
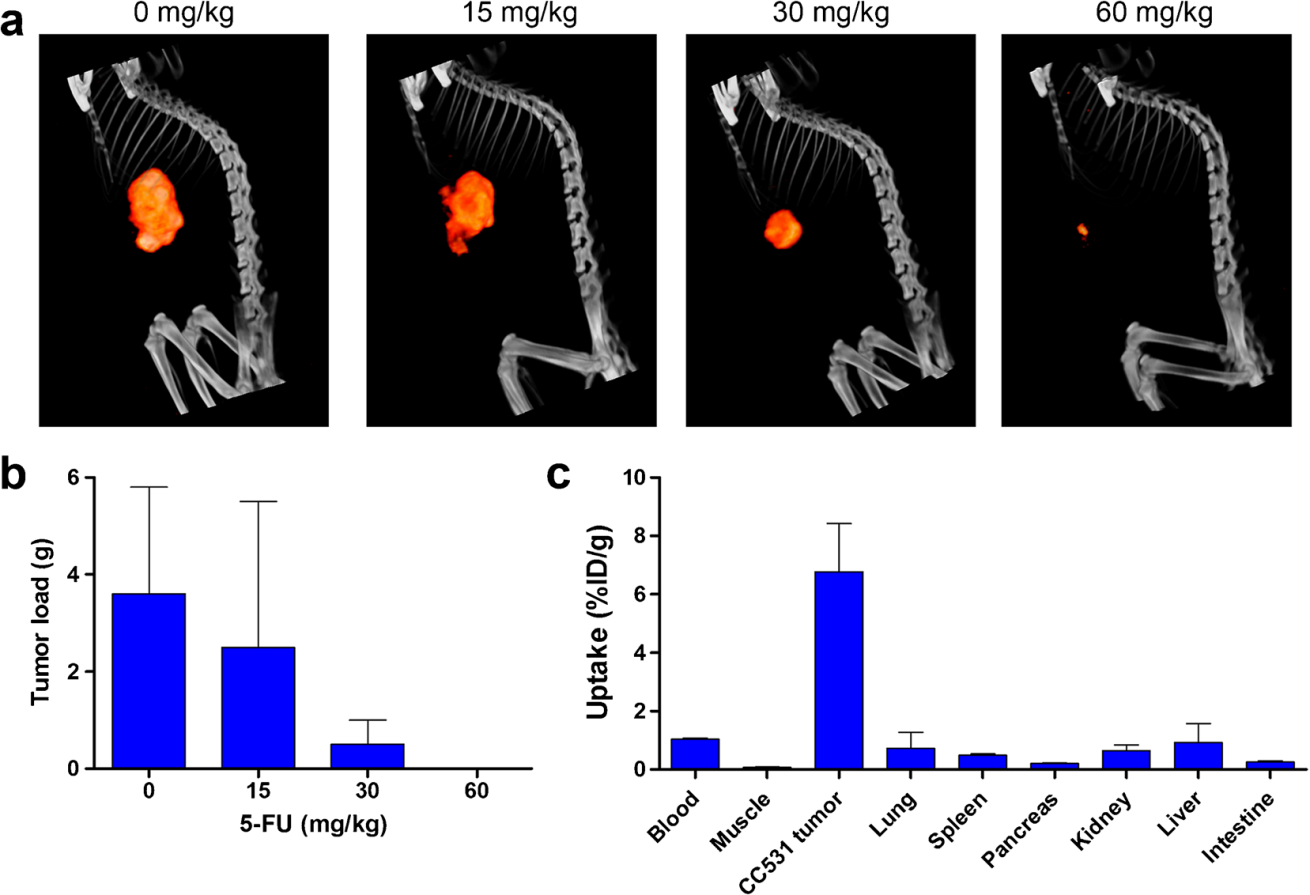
**a** Representative SPECT/CT images of rats with CC531 tumors, acquired at day 19 after start of 5-FU treatment. **b** CC531 tumor load after four cycles of 5-FU treatment as measured after dissection of the tumors presented as mean ± SD (*n* = 5 per group). **c** Biodistribution of [^111^In]MG1 after dissection presented as mean ± SD (*n* = 5 per group).

**Fig. 3.**
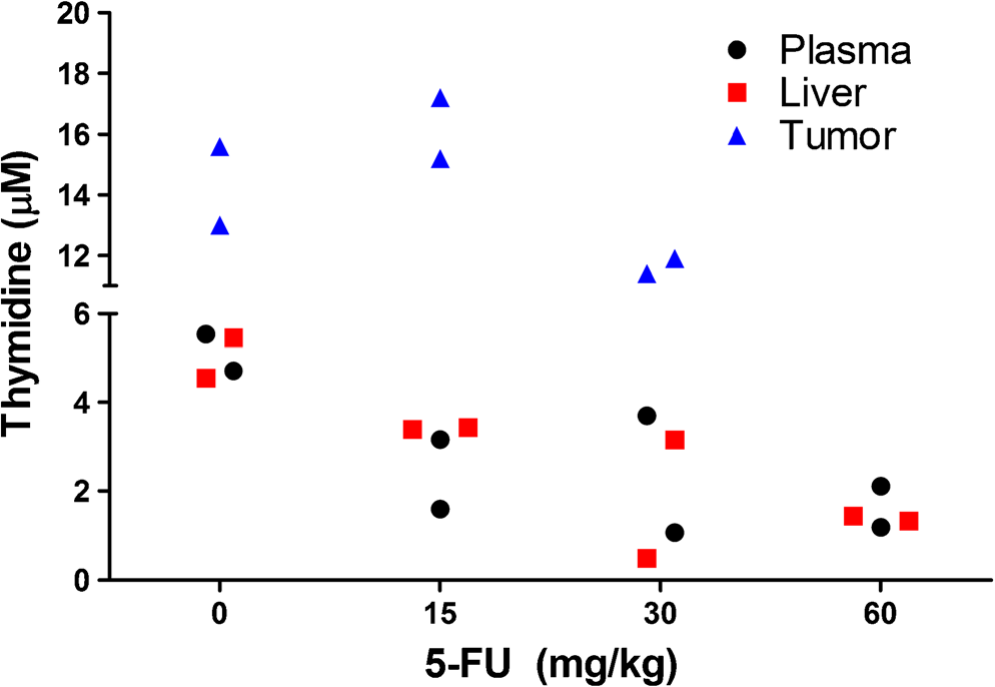
Thymidine levels in plasma, liver, and tumor as measured by LC-MS. Rats treated with 60 mg/kg 5-FU did not have any residual tumor to analyze tumor thymidine levels.

To determine the optimal 5-FU dose for treatment of CC531 liver tumors, four groups of Wag/Rij rats with liver tumors were treated with vehicle, 15 mg/kg, 30 mg/kg, or 60 mg/kg 5-FU. After treatment, rats were scanned by SPECT/CT to determine tumor load after treatment. Examples of SPECT/CT scans acquired 19 days after the start of treatment are presented in Fig. 2a. After four cycles of 5-FU, rats were euthanized and tumors were excised. Macroscopic tumor growth was absent in 5/5, 1/5, 2/5, and 0/5 rats treated with 60 mg/kg, 30 mg/kg, 15 mg/kg 5-FU, and vehicle only, respectively. The mean weights of macroscopically visible tumors are presented in Fig. 2b and the *ex vivo* biodistribution of [^111^In]MG1 is presented in Fig. 2c. Tumor load was significantly decreased in the 30 and 60 mg/kg groups, compared to the rats that received vehicle only (*p* < 0.012). Thymidine levels in plasma and liver decreased with increasing doses of 5-FU, while there was no major decrease in tumor thymidine levels (Fig. 3). In the 60 mg/kg group, no residual tumor tissue was left to analyze thymidine levels.

### Immunohistochemical Analysis of CC531 Liver Tumors

**Fig. 4.**
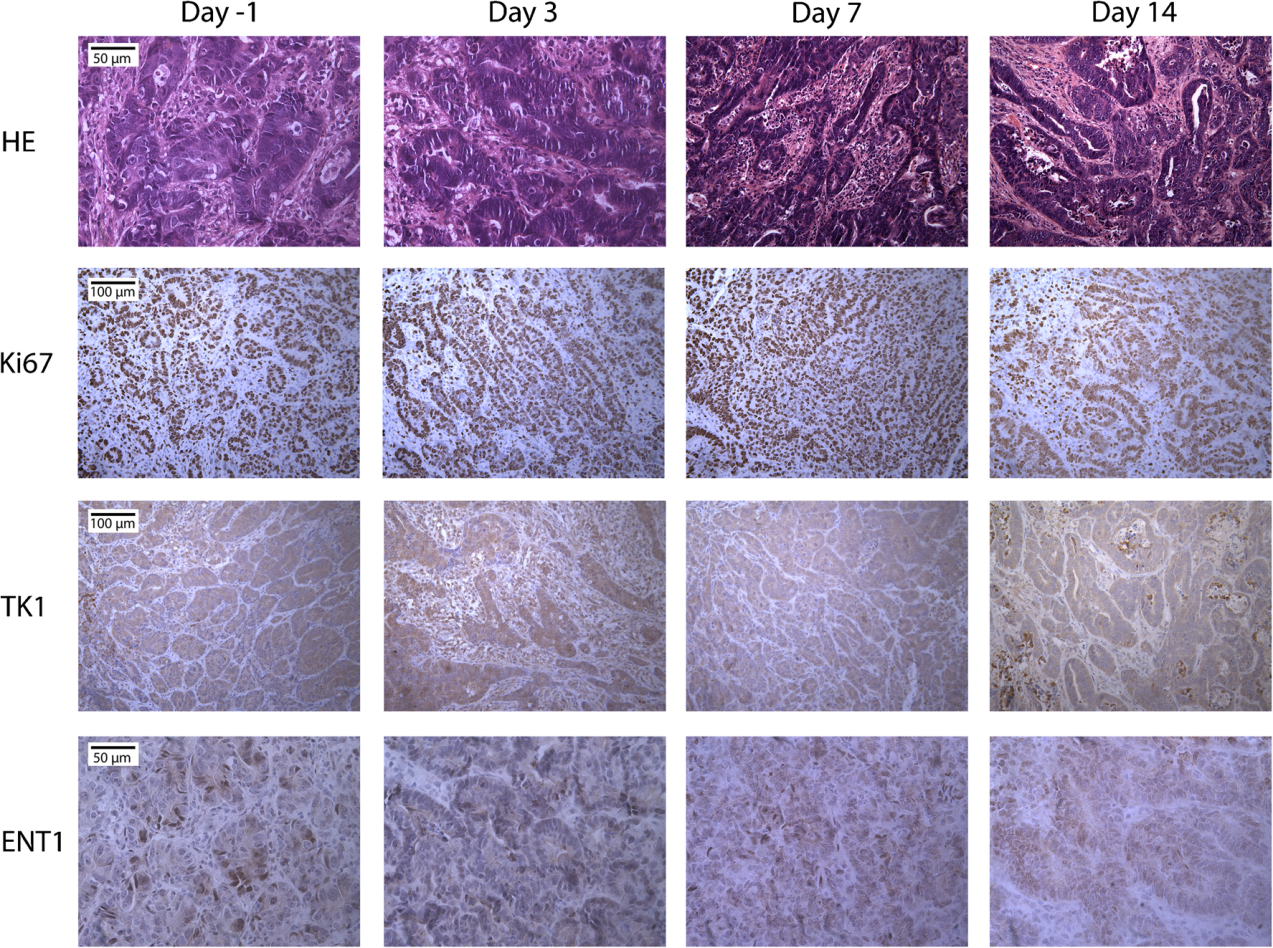
Immunohistochemical analysis of CC531 liver tumors treated with 60 mg/kg 5-FU. Magnification: HE and ENT1 ×400, Ki67 and TK1 ×200.

CC531 tumor sections were analyzed for necrosis, Ki67, TK1, and ENT1 expression. Representative images of 5-FU-treated tumors are presented in Fig. 4. HE staining showed an increase in necrotic areas in both control and 5-FU-treated tumors. The percentage of Ki67 positive tumor cells did not change during treatment and was 86 ± 7, 92 ± 4, 90 ± 4, 89 ± 5, and 78 ± 4 %, for tumors at days −1, 1, 3, 7, and 14 post-5-FU treatment, respectively. TK1 and ENT1 staining did not show obvious changes between vehicle and 5-FU-treated rats.

### 5-FU Treatment Induces a Flare of [^18^F]FLT Uptake in CC531 Liver Tumors

**Fig. 5.**
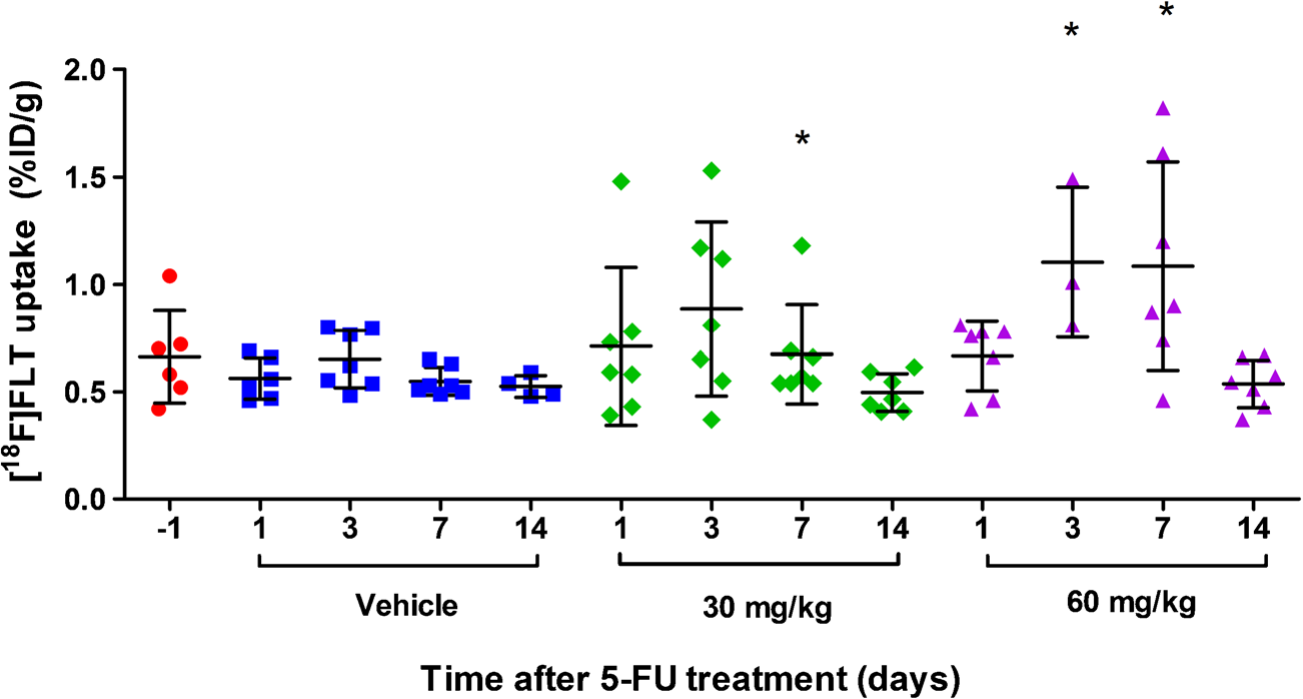
Uptake of [^18^F]FLT in CC531 tumors after 5-FU treatment (mean ± SD). Uptake was measured after dissection of the tumors. *Asterisks* indicate a significant difference in the mean [^18^F]FLT uptake in 5-FU-treated tumors compared with the vehicle-treated tumors at the same time point. The *symbols* represent the individual rats.

While conventional immunohistochemical biomarkers did not correlate with 5-FU treatment response, [^18^F]FLT uptake in CC531 tumors increased significantly during 5-FU therapy. Biodistribution studies showed that tumor uptake of [^18^F]FLT increased at days 3 and 7 after treatment with 60 mg/kg 5-FU (*p* = 0.017 and 0.026, respectively), compared with [^18^F]FLT tumor uptake measured at the same time point in the control group (Fig. 5). There was no clear increase in [^18^F]FLT uptake in the 30 mg/kg group. At day 7, [^18^F]FLT tumor uptake did significantly differ compared with untreated tumors (*p* = 0.038). This could be explained by one single outlier in this group.

**Fig. 6.**
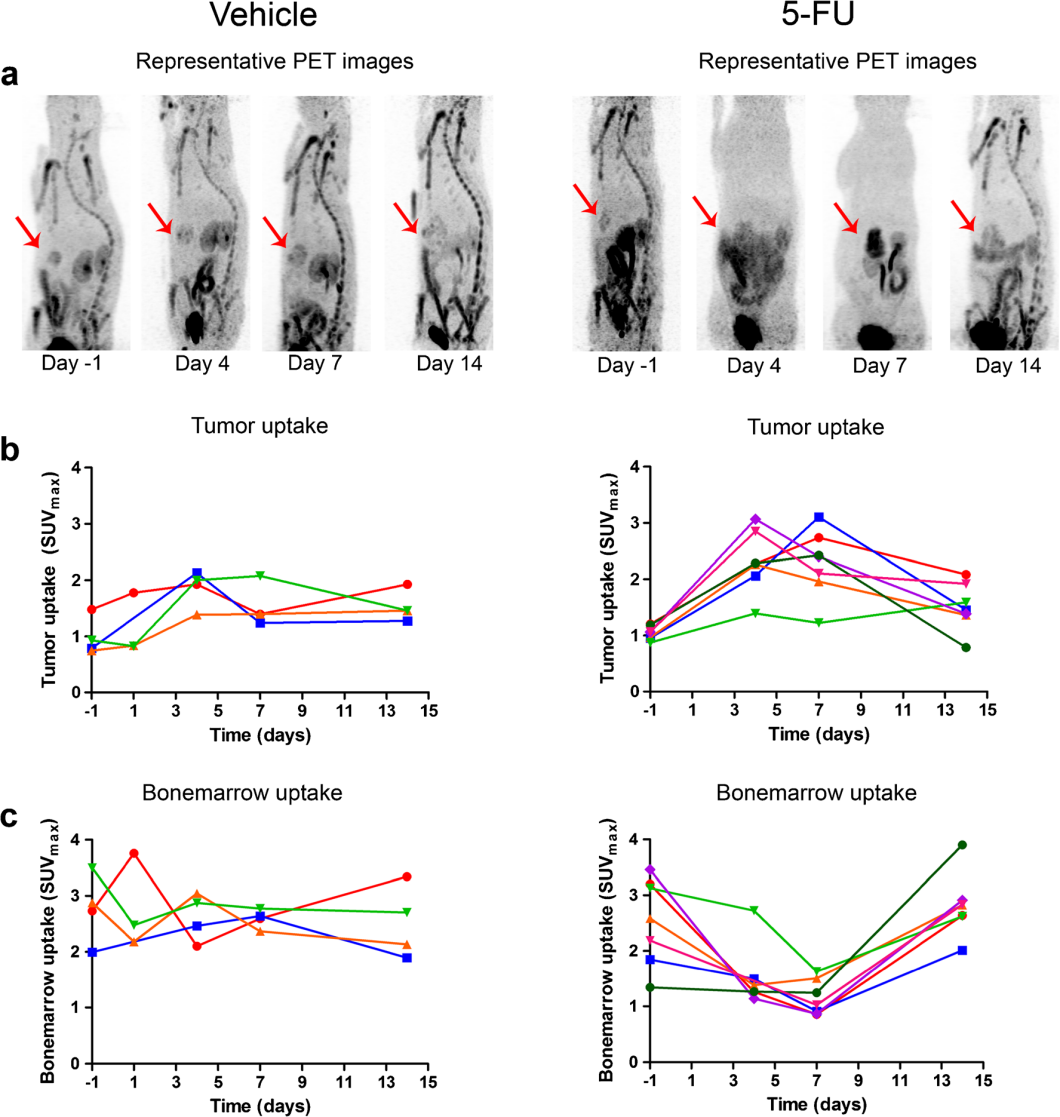
**a** Representative [^18^F]FLT PET scans of rats treated with vehicle or 60 mg/kg 5-FU. Tumors are indicated by the *red arrows*. The [^18^F]FLT uptake was quantified for **b** tumor and **c** femur. The *lines* represent individual rats.

In a separate group of animals (*n* = 7), intrahepatic tumors were monitored longitudinally with [^18^F]FLT PET. In six out of seven rats, a more than 2-fold increase in [^18^F]FLT SUV_max_ was observed at day 4 or 7 posttreatment, while for one out of seven rats, the maximum increase in [^18^F]FLT SUV_max_ was obtained at day 14 posttreatment (1.6-fold increase). Figure 6a shows typical examples of [^18^F]FLT PET scans of a 5-FU-treated rat and vehicle-treated rat. Quantification of [^18^F]FLT scans (Fig. 6b, c) showed that the SUV_mean_ (data not shown) and SUV_max_ were increased at days 4 and 7 after 60 mg/kg 5-FU. SUV_max_ at baseline, and 4, 7, and 14 days after treatment was 1.05 ± 0.12, 2.31 ± 0.54, 2.28 ± 0.60, and 1.51 ± 0.42, respectively (*p* = 0.002). In the control group, SUV_mean_ and SUV_max_ did not change significantly. The [^18^F]FLT uptake in bone marrow was significantly decreased after 5-FU treatment (*p* = 0.004) and was not affected in the vehicle group.

### 5-FU Treatment Does not Cause Changes in the ADC of CC531 Liver Tumors

**Fig. 7.**
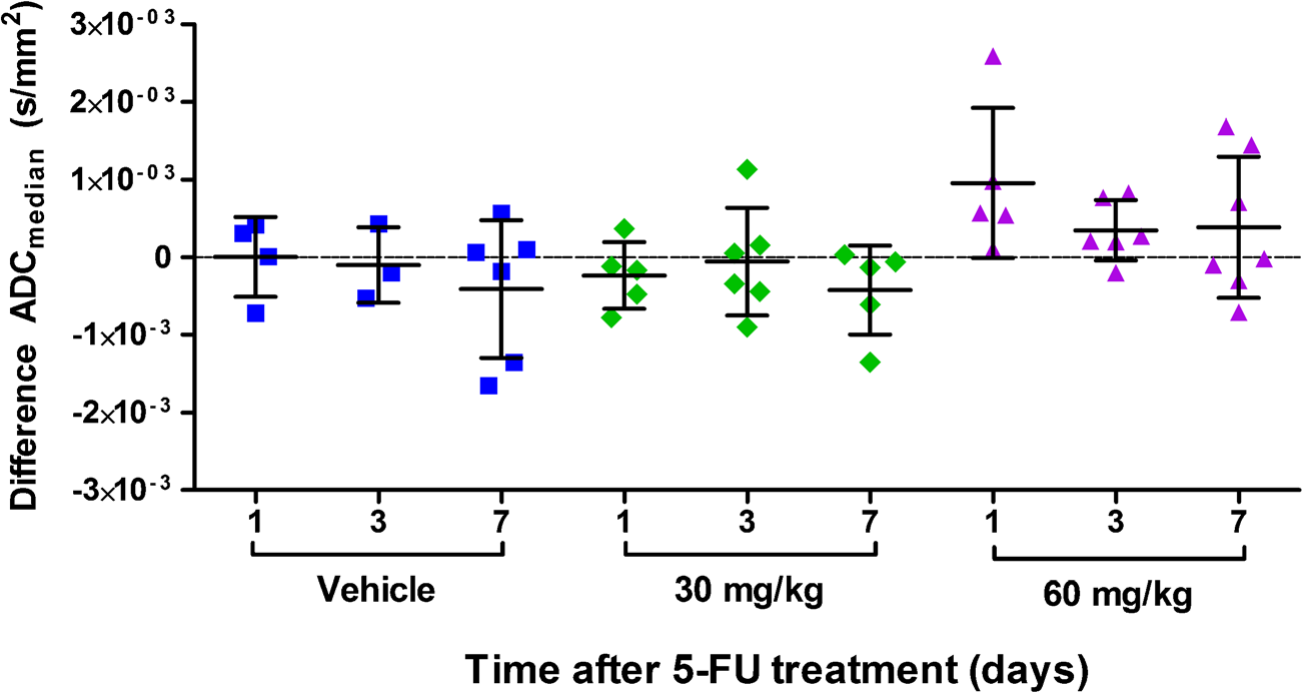
Difference in ADC_mean_ of CC531 tumors treated with vehicle or 5-FU, compared with pretreatment scan. The *symbols* represent the individual rats.

The pretreatment and posttreatment ADC_median_ values are depicted in Fig. 7 and Table 1. The ADC seems to increase slightly at day 1 after 60 mg/kg 5-FU. However, the change in ADC_median_, ADC_mean_, and 75th and 25th percentiles in 5-FU-treated groups was not significantly different compared to the change in vehicle-treated groups. There was no correlation between the change in ADC_median_ and [^18^F]FLT uptake.

**Table 1:** ADC_median_ of CC531 tumors treated with vehicle or 5-FU

Treatment	ADC_median_ (×10^−3^ mm^2^/s)	
	Baseline	Posttreatment
Vehicle		
Day 1	1.18 ± 0.40	1.17 ± 0.35
Day 3	1.74 ± 0.51	1.64 ± 0.38
Day 7	1.59 ± 0.84	1.19 ± 0.11
5-FU (30 mg/kg)		
Day 1	1.38 ± 0.55	1.15 ± 0.36
Day 3	1.37 ± 0.45	1.18 ± 0.55
Day 7	1.40 ± 0.53	1.04 ± 0.38
5-FU (60 mg/kg)		
Day 1	1.47 ± 0.37	2.11 ± 0.96
Day 3	1.55 ± 1.04	1.57 ± 0.74
Day 7	1.13 ± 0.42	1.51 ± 0.60

## Discussion

The aim of this study was to evaluate whether the observed therapeutic effect of 5-FU could be monitored with DW-MRI and [^18^F]FLT PET in a syngeneic rat model of CC531 liver tumors. It seems that 5-FU did not induce significant changes in ADC_median_ compared with vehicle-treated rats. In contrast, a flare effect was observed for [^18^F]FLT PET at days 3 to 7 after start of treatment. At later time points, [^18^F]FLT uptake seemed to return to baseline.

DW-MRI can be used to characterize cellular integrity and chemotherapy-induced cell death [6]. Early 5-FU therapy effects can induce cell swelling, resulting in a decreased ADC, while at later time points, apoptosis and necrosis can occur, resulting in an increased ADC. In CC531 liver tumors, we did not observe an increase in ADC during 5-FU treatment. Potentially, the ADC changes at days 3 and 7 could have been masked by cellular swelling early after start of therapy. Tumor necrosis was analyzed immunohistochemically. The increase in necrosis did not differ between 5-FU-treated and control tumors. Other factors which correlate to ADC which warrant future research are cell density, extracellular space, and nuclear-to-cytoplasmic ratio [6]. A few clinical studies have shown an increase in ADC upon arterial infusion of 5-FU or chemoradiation [19–21]. These ADC measurements were performed in patients treated for at least 1 week or longer with 5-FU, while in our study, rats were only measured up to 7 days posttreatment. In addition, most clinical studies were performed in patients receiving chemoradiation, and this combination may have a more pronounced effect on tumor ADC than 5-FU alone.

In this study, we observed a significant increase in [^18^F]FLT during 5-FU treatment. In clinical practice, 5-FU is frequently administered in combination with oxaliplatin. *In vitro*, we have shown that the addition of oxaliplatin did not affect the [^18^F]FLT flare. The uptake of [^18^F]FLT has been linked to cellular proliferation; however, [^18^F]FLT is not incorporated into the DNA [10]. Its accumulation depends on activity of the ENT1 transporter, the rate of phosphorylation by TK1, and the level of endogenous thymidine [15, 22, 23]. Therefore, [^18^F]FLT uptake does not necessarily reflect tumor cell proliferation. For example, drugs that inhibit the de novo pathway of DNA synthesis can alter the activity of TK1, and thus that of [^18^F]FLT phosphorylation and its trapping in the tumor cell [24]. 5-FU is an antimetabolite that inhibits thymidylate synthase (TS), which is a critical enzyme in the *de novo* synthesis of thymidine. As a consequence, tumor cells can upregulate the thymidine salvage pathway, resulting in increased TK1 activity and redistribution of ENT1 to the cell surface, which leads to enhanced [^18^F]FLT uptake and trapping in the tumor cell. Here, we indeed observed a flare in [^18^F]FLT uptake after 5-FU treatment. A potential explanation might be an increase in TK1 and ENT1 expression and/or activity induced by 5-FU treatment. Immunohistochemical analysis of our tumor samples did not show a clear upregulation of TK1 or ENT1 expression. However, it must be noted that differences in enzyme activity or distribution at the cell surface cannot be assessed by these immunohistochemical analyses. Literature reports contrasting information regarding TK1 and ENT1. Some studies describe an increase in TK1 activity and redistribution of ENT1 to the cell membrane, while others show that 5-FU does not alter TK1 and/or ENT1 expression or activity [23–31]. Another possible explanation for the increase in [^18^F]FLT uptake might be changes in the level of endogenous thymidine. We observed decreased levels of endogenous thymidine levels in plasma and liver after 4 weeks of 5-FU treatment. Endogenous thymidine and [^18^F]FLT compete for tumor cell uptake via ENT1 and phosphorylation by TK1 [23, 32]. However, further research is needed to assess whether this indeed caused the flare of [^18^F]FLT uptake at earlier time points after start of treatment. Overall, our data indicate that [^18^F]FLT uptake does not solely reflect tumor cell proliferation but that the pathway responsible for [^18^F]FLT uptake is more complex.

A few clinical studies have assessed the role of [^18^F]FLT PET for early response monitoring during 5-FU-containing treatment regimens. In two studies, patients with rectal cancer were scanned before and 2 weeks after chemoradiation. [^18^F]FLT uptake decreased significantly, but did not correlate to histopathological tumor regression [33, 34]. However, a strong decrease in [^18^F]FLT uptake (decrease in SUV_max_ ≥ 60 %) was associated with prolonged disease-free survival [34]. Hong *et al.* analyzed [^18^F]FLT uptake 1 and 3 days after 5-FU infusion in a FOLFOX-treatment regimen. [^18^F]FLT flare 1 day after 5-FU administration was related to poor treatment response as assessed by CT. Three days after treatment, [^18^F]FLT uptake was significantly decreased in responders, while it was not different from the baseline scan in nonresponders. This study excluded liver metastases from analysis [35].

In the current study, we found a flare in [^18^F]FLT uptake in 5-FU responsive liver CC531 liver tumors in rats. This is in contrast with the abovementioned studies. However, it must be noted that none of these studies included liver metastases in their analysis. In clinical studies, [^18^F]FLT PET imaging of hepatic metastases is hampered by the increased retention of [^18^F]FLT in the liver, reducing the sensitivity. In addition, timing of [^18^F]FLT follow-up scan appears of crucial importance to measure the flare effect. In human xenografts in mice, it has been reported that [^18^F]FLT flare was detectable as early as 1 h to 1 day posttreatment, while in our experiments, this was detectable 4 to 7 days posttreatment [24, 25, 29]. The only clinical study that included measurements 1 to 3 days after 5-FU treatment indeed showed an increase in [^18^F]FLT uptake, although this was associated with a poor response instead of good response [35].

In contrast to the increased [^18^F]FLT uptake in CC531 tumors, a decrease in [^18^F]FLT uptake was observed in bone marrow of 5-FU-treated rats. [^18^F]FLT uptake in bone marrow is caused by high proliferative activity of hematopietic cells. Chemotherapeutic drugs inhibit the proliferation of these cells and may thus reduce the [^18^F]FLT uptake. In our model, we showed that 5-FU has differential effects on proliferating bone marrow cells compared with CC531 tumor cells. To our knowledge, there are only few studies that report on [^18^F]FLT uptake in bone marrow after treatment with TS inhibitors. One study in breast cancer patients treated with pemetrexed showed enhanced [^18^F]FLT bone marrow uptake 4 h after treatment, while tumor uptake did not change [36]. Thus, the flare in [^18^F]FLT uptake in bone marrow might occur at earlier time points then measured in our study. Other studies with TS inhibitors have also reported differential effects on [^18^F]FLT uptake in tumor and normal proliferating tissues, suggesting that bone marrow may respond differently to TS inhibitors than tumor cells [37]. Future research is warranted to investigate the role of TK1 and ENT1 in this.

This study suggests that 5-FU does not affect ADC but induces a flare in [^18^F]FLT uptake in CC531 liver tumors. However, several limitations should be taken into account when interpreting these data. First of all, the reproducibility of the ADC and [^18^F]FLT measurements in this specific model is unknown and this should be addressed in future studies. Second, some of the experimental groups in the study consisted of a small number of animals (three to seven rats per group). Several rats had to be excluded from the study because of lack of intrahepatic tumor growth. Finally, ADC measurements in rat liver are very sensitive to motion artifacts due to breathing of the animal. Although respiratory gating was applied, this might still have had a negative impact on image quality.

## Conclusion

This study suggests that 5-FU treatment induces a flare in [^18^F]FLT uptake of responsive CC531 tumors in the liver, while the ADC did not change significantly. An increase in [^18^F]FLT PET uptake might predict response to treatment, although accurate timing is crucial to identify and measure the [^18^F]FLT flare effect. Additional studies in larger groups are warranted to investigate the reproducibility of the [^18^F]FLT and ADC measurements in this model. Furthermore, it remains to be determined whether [^18^F]FLT PET can discriminate between disease progression and treatment response in preclinical cancer models. Finally, studies in colorectal cancer patients with liver metastasis are warranted, to determine whether this flare effect can also be measured in patients treated with 5-FU.

## Electronic supplementary material

ESM 1 (PDF 121 kb)

## References

[1] Hurwitz H, Fehrenbacher L, Novotny W (2004). Bevacizumab plus irinotecan, fluorouracil, and leucovorin for metastatic colorectal cancer. N Engl J Med.

[2] Jonker DJ, O’Callaghan CJ, Karapetis CS (2007). Cetuximab for the treatment of colorectal cancer. N Engl J Med.

[3] Van Cutsem E, Nordlinger B, Cervantes A, Group EGW (2010). Advanced colorectal cancer: ESMO Clinical Practice Guidelines for treatment. Ann Oncol.

[4] Paproski RJ, Ng AM, Yao SY (2008). The role of human nucleoside transporters in uptake of 3′-deoxy-3′-fluorothymidine. Mol Pharmacol.

[5] Soloviev D, Lewis D, Honess D, Aboagye E (2012). [(18)F]FLT: an imaging biomarker of tumour proliferation for assessment of tumour response to treatment. Eur J Cancer.

[6] Sinkus R, Van Beers BE, Vilgrain V (2012). Apparent diffusion coefficient from magnetic resonance imaging as a biomarker in oncology drug development. Eur J Cancer.

[7] Zedeck MS, Sternberg SS (1974). A model system for studies of colon carcinogenesis: tumor induction by a single injection of methylazoxymethanol acetate. J Natl Cancer Inst.

[8] Koppe MJ, Hendriks T, Boerman OC (2006). Radioimmunotherapy is an effective adjuvant treatment after cytoreductive surgery of experimental colonic peritoneal carcinomatosis. J Nucl Med.

[9] Hamacher K, Coenen HH, Stocklin G (1986). Efficient stereospecific synthesis of no-carrier-added 2-[18F]-fluoro-2-deoxy-D-glucose using aminopolyether supported nucleophilic substitution. J Nucl Med.

[10] Buck AK, Halter G, Schirrmeister H (2003). Imaging proliferation in lung tumors with PET: 18F-FLT versus ^18^F-FDG. J Nucl Med.

[11] Hagenaars M, Koelemij R, Ensink NG (2000). The development of novel mouse monoclonal antibodies against the CC531 rat colon adenocarcinoma. Clin Exp Metastasis.

[12] Heskamp S, van Laarhoven HW, Molkenboer-Kuenen JD (2010). ImmunoSPECT and immunoPET of IGF-1R expression with the radiolabeled antibody R1507 in a triple-negative breast cancer model. J Nucl Med.

[13] de Jong GM, Hendriks T, Eek A (2011). Adjuvant radioimmunotherapy improves survival of rats after resection of colorectal liver metastases. Ann Surg.

[14] de Jong GM, Hendriks T, Eek A (2009). Radioimmunotherapy improves survival of rats with microscopic liver metastases of colorectal origin. Ann Surg Oncol.

[15] Schelhaas S, Wachsmuth L, Viel T (2014). Variability of proliferation and diffusion in different lung cancer models as measured by 3′-deoxy-3′-18F-fluorothymidine PET and diffusion-weighted MR imaging. J Nucl Med.

[16] Heinzmann K, Honess DJ, Lewis DY (2016). The relationship between endogenous thymidine concentrations and [(18)F]FLT uptake in a range of preclinical tumour models. Eur J Nucl Med Mol Imaging Res.

[17] Disselhorst JA, Brom M, Laverman P (2010). Image-quality assessment for several positron emitters using the NEMA NU 4-2008 standards in the Siemens Inveon small-animal PET scanner. J Nucl Med.

[18] Boellaard R, O’Doherty MJ, Weber WA (2010). FDG PET and PET/CT: EANM procedure guidelines for tumour PET imaging: version 1.0. Eur J Nucl Med Mol Imaging.

[19] Marugami N, Tanaka T, Kitano S (2009). Early detection of therapeutic response to hepatic arterial infusion chemotherapy of liver metastases from colorectal cancer using diffusion-weighted MR imaging. Cardiovasc Intervent Radiol.

[20] Kremser C, Judmaier W, Hein P, Griebel J, Lukas P, de Vries A (2003). Preliminary results on the influence of chemoradiation on apparent diffusion coefficients of primary rectal carcinoma measured by magnetic resonance imaging. Strahlentherapie Onkologie.

[21] Lambrecht M, Vandecaveye V, De Keyzer F (2012). Value of diffusion-weighted magnetic resonance imaging for prediction and early assessment of response to neoadjuvant radiochemotherapy in rectal cancer: preliminary results. Int J Radiat Oncol Biol Phys.

[22] Shields AF (2012). PET imaging of tumor growth: not as easy as it looks. Clin Cancer Res.

[23] Zhang CC, Yan Z, Li W (2012). [(18)F]FLT-PET imaging does not always "light up" proliferating tumor cells. Clin Cancer Res.

[24] Lee SJ, Kim SY, Chung JH (2010). Induction of thymidine kinase 1 after 5-fluorouracil as a mechanism for 3′-deoxy-3′-[^18^F]fluorothymidine flare. Biochem Pharmacol.

[25] Hong IK, Kim SY, Chung JH (2014). 3′-Deoxy-3′-[^18^F]fluorothymidine positron emission tomography imaging of thymidine kinase 1 activity after 5-fluorouracil treatment in a mouse tumor model. Anticancer Res.

[26] Dittmann H, Dohmen BM, Kehlbach R (2002). Early changes in [^18^F]FLT uptake after chemotherapy: an experimental study. Eur J Nucl Med Mol Imaging.

[27] Plotnik DA, McLaughlin LJ, Krohn KA, Schwartz JL (2012). The effects of 5-fluoruracil treatment on 3′-fluoro-3′-deoxythymidine (FLT) transport and metabolism in proliferating and non-proliferating cultures of human tumor cells. Nucl Med Biol.

[28] Kameyama R, Yamamoto Y, Izuishi K (2011). Correlation of 18F-FLT uptake with equilibrative nucleoside transporter-1 and thymidine kinase-1 expressions in gastrointestinal cancer. Nucl Med Commun.

[29] Perumal M, Pillai RG, Barthel H (2006). Redistribution of nucleoside transporters to the cell membrane provides a novel approach for imaging thymidylate synthase inhibition by positron emission tomography. Cancer Res.

[30] Pillai RG, Forster M, Perumal M (2008). Imaging pharmacodynamics of the alpha-folate receptor-targeted thymidylate synthase inhibitor BGC 945. Cancer Res.

[31] Barthel H, Cleij MC, Collingridge DR (2003). 3′-deoxy-3′-[18F]fluorothymidine as a new marker for monitoring tumor response to antiproliferative therapy in vivo with positron emission tomography. Cancer Res.

[32] McKinley ET, Ayers GD, Smith RA (2013). Limits of [^18^F]-FLT PET as a biomarker of proliferation in oncology. PLoS One.

[33] Wieder HA, Geinitz H, Rosenberg R (2007). PET imaging with [^18^F]3′-deoxy-3′-fluorothymidine for prediction of response to neoadjuvant treatment in patients with rectal cancer. Eur J Nucl Med Mol Imaging.

[34] Dehdashti F, Grigsby PW, Myerson RJ (2013). Positron emission tomography with [^18^F]-3′-deoxy-3’fluorothymidine (FLT) as a predictor of outcome in patients with locally advanced resectable rectal cancer: a pilot study. Mol Imaging Biol.

[35] Hong YS, Kim HO, Kim KP (2013). 3′-Deoxy-3′-18F-fluorothymidine PET for the early prediction of response to leucovorin, 5-fluorouracil, and oxaliplatin therapy in patients with metastatic colorectal cancer. J Nucl Med.

[36] Frings V, van der Veldt AA, Boellaard R (2013). Pemetrexed induced thymidylate synthase inhibition in non-small cell lung cancer patients: a pilot study with 3′-deoxy-3′-[^18^F]fluorothymidine positron emission tomography. PLoS One.

[37] Kenny LM, Contractor KB, Stebbing J (2009). Altered tissue 3′-deoxy-3′-[^18^F]fluorothymidine pharmacokinetics in human breast cancer following capecitabine treatment detected by positron emission tomography. Clin Cancer Res.

