# Supplementary material for: Response Monitoring with [18F]FLT PET and Diffusion-Weighted MRI After Cytotoxic 5-FU Treatment in an Experimental Rat Model for Colorectal Liver Metastases
Source: Mol Imaging Biol. 2016 Oct 31;19(4):540–9. doi: 10.1007/s11307-016-1021-2 (PMC5498638; doi:10.1007/s11307-016-1021-2)
Supplement: Supplementary file 1 — (PDF 121 kb) [file 11307_2016_1021_MOESM1_ESM.pdf]

Electronic Supplementary Material

Response Monitoring with [^{18}F]FLT PET and Diffusion Weighted MRI after Cytotoxic 5-FU Treatment in an Experimental Rat Model for Colorectal Liver Metastases

Journal: Molecular Imaging and Biology

Sandra Heskamp[1], Linda Heijmen[1], Danny Gerrits[1], Janneke D.M. Molkenboer-Kuenen[1], Edwin G.W. ter Voert[1], Kathrin Heinzmann[2], Davina J. Honess[2], Donna-Michelle Smith[2], John R. Griffiths[2], Sabrina Doblas[3], Ralph Sinkus[4], Peter Laverman[1], Wim J.G. Oyen[1], Arend Heerschap[1], Otto C. Boerman[1]

[1] Department of Radiology and Nuclear Medicine, Radboud University Medical Center, Nijmegen, The Netherlands

[2] Cancer Research UK Cambridge Institute, University of Cambridge, Cambridge, UK

[3] LBI, CRI – UMR 1149 Inserm, Université Paris Diderot, Paris, France

[4] BHF Centre of Excellence, Division of Imaging Sciences and Biomedical Engineering, King's College London, King's Health Partners, St. Thomas' Hospital, London, SE1 7EH, United Kingdom

Corresponding author:

Sandra Heskamp
Radboud university medical center
Department of Radiology and Nuclear Medicine (Internal Postal Code 756)
P.O. Box 9101, 6500 HB Nijmegen, The Netherlands
Phone: +31243619097, Fax: +31243618942
E-mail: sandra.heskamp@radboudumc.nl

Supplemental Methods

SPECT/CT

SPECT/CT scans were acquired with the U-SPECT-II/CT (MILabs, Utrecht, The Netherlands). Rats were scanned under general anesthesia (isoflurane/O_2) for 50 min using the 1.0 mm diameter rat collimator tube, followed by a CT scan (spatial resolution 160 µm, 65 kV, 615 µA) for anatomical reference. Scans were reconstructed with MILabs reconstruction software, using an ordered-subset expectation maximization algorithm, with a voxel size of 0.75 mm. SPECT/CT scans were analyzed and maximum intensity projections (MIPs) were created using the Inveon Research Workplace software (IRW, version 4.1).

DW-MRI

DW-MRI was performed at 11.7T on a Bruker Biospec MR system under Paravision 5.1 using a Bruker Mouse Body Volume Coil. The outer diameter of the coil is 75 mm and the inner diameter is 40 mm, which is suited to fit small rats. Anesthesia was induced with 4% isoflurane and rats were maintained under anesthesia with 2% isoflurane, the respiratory rate of the rats was monitored and kept constant at approximately 40 breaths/min and temperature was maintained at 37 °C. For morphological assessment, a T2-weighted MR image was acquired with slices covering the whole tumor area. The slice with the largest tumor diameter was chosen for DW MRI. DW MRI acquisition was respiratory gated and followed a spin-echo EPI scheme with fat suppression, TE/TR of 16/2000 ms, 4 averages, 23 segments, b-values 0, 150, 300 and 600 s/mm^2, slice thickness 2 mm, FoV 5 cm, matrix 128x128. ADC maps were calculated (using b = 0, 150, 300 and 600 s/mm^2 images) by fitting a monoexponential model with the ISA-tool on the Bruker scanner software and were exported as dicom files. Tumor ROIs were defined manually on the b = 0 images and then copied on the ADC maps using IRW v4.1. Data per voxel were exported to excel in order to facilitate histogram analyses. ADC_{mean}, ADC_{median}, 25th and 75th percentile values were calculated.

[^{18}F]FLT PET

[^{18}F]FLT PET scans were acquired using the Inveon PET/CT system (Siemens Preclinical Solutions, Knoxville, TN) with an intrinsic spatial resolution of 1.5 mm.*(1)* Rats were injected intravenously with 10-

12 MBq [^{18}F]FLT and 50 min post injection, anesthesia was induced with 4% isoflurane in air. Rats were maintained under anesthesia with 2% isoflurane. At 60 min post injection, a PET emission scan was acquired for 15 min (60 – 75 min p.i.), followed by a ^{57}Co transmission scan of 3 min. Scans were reconstructed using the Inveon Acquisition Workplace software (version 1.5, Siemens Preclinical Solutions), using an ordered-set expectation maximization 3-dimensional maximum a posteriori algorithm with the following parameters: matrix, 256 x 256 x 159, pixel size, 0.43 x 0.43 x 0.8 mm^3, and β-value of 1.5 with uniform variance. Images were analyzed using IRW v4.1. A 3D isocontour was set at 41% of the maximum pixel value adapted for background (normal liver) to determine pre- and posttreatment SUV_{mean} and SUV_{max}.*(2)* To determine the SUV_{max} in bone marrow, a 3D volume of interest was drawn around the left femur and subsequently, an isocontour was set at 50% of the maximum pixel value.

Immunostaining

Antigen retrieval was performed for 10 min at 99 °C in 10 mM sodium citrate pH 6.0 (Ki67 and TK1) or 10 mM TRIS, 1 mM EDTA, 0.05% Tween, pH 9.0 (ENT1). Endogenous peroxidase activity was blocked with 3% H_2O_2 and nonspecific binding was blocked by incubation with normal swine serum (Ki67 and TK1) or normal goat serum and avidin/biotin blocking (SP-2001, Vector, ENT1). After incubation with the primary antibody, Ki67 and TK1-stained tumor sections were incubated with a peroxidase-conjugated secondary antibody and ENT1-stained sections with a biotinylated secondary antibody, followed by incubation with an avidin-biotin-enzyme complex (Vector Laboratories, Burlingame, CA). Finally 3,3'-Diaminobenzidine (DAB) was used to develop the staining of the tumor sections.

References

1. Disselhorst JA, Brom M, Laverman P, et al. (2010) Image-quality assessment for several positron emitters using the NEMA NU 4-2008 standards in the Siemens Inveon small-animal PET scanner. J Nucl Med. 51:610-617.
2. Boellaard R, O'Doherty MJ, Weber WA, et al. (2010) FDG PET and PET/CT: EANM procedure guidelines for tumour PET imaging: version 1.0. Eur J Nucl Med Mol Imaging 37:181-200.
